# Effects of Neutral Postures on Mechanical Properties of Cervical Spine Under Different Gravitational Environments: A Musculoskeletal Model Study

**DOI:** 10.3390/life15030447

**Published:** 2025-03-12

**Authors:** Zhanyang He, Bin Zhang, Binyong Ye, Zhanbing Song, Qiang Mei, Jiahao Xu, Houwei Zhu

**Affiliations:** 1 College of Physical Education and Health Sciences, Zhejiang Normal University, Jinhua 321000, China; 2908161568@zjnu.edu.cn (Z.H.); zhangbin666@zjnu.edu.cn (B.Z.); binyongye@163.com (B.Y.); 201931070018@mail.bnu.edu.cn (Z.S.); 2209569951@zjnu.edu.cn (Q.M.); x19511319782@163.com (J.X.); 2 Sports Coaching College, Beijing Sport University, Beijing 100084, China

**Keywords:** space flight, microgravity, cervical injury, intervertebral disc injury, musculoskeletal model

## Abstract

This study used the Anybody musculoskeletal model to investigate the effects of different neutral postures on the cervical spine and its associated muscle mechanical properties in various gravitational environments. A full-body musculoskeletal model (male, height: 1.74 m, mass: 74 kg) from the AMMR database, developed using the Anybody Modeling System, was employed to perform a quantitative analysis of three postures, including the neutral body posture in microgravity (NBP 0G), the neutral body posture in normal gravity (NBP 1G), and the relaxed standing posture in microgravity (SM 0G). The results showed that, compared to the NBP 1G posture in a gravitational environment, adopting the NBP 0G posture in microgravity resulted in an average reduction of 76.6% in the compressive force of the intervertebral discs, with shear forces in the same direction decreasing by 7.97 to 12.57 N. The shear force direction at the C6–C7 and C7–T1 segments changed, the intervertebral disc height increased by 1.6–4.8%, the disc cross-sectional area expanded by 3.2–6.9%, and the disc volume expanded by 4.8–9.4%. In addition, the total muscle force at the cervical region decreased while the ligament force increased. These changes in mechanical properties may significantly increase the risk of cervical disc herniation and degenerative disc diseases, as well as the risk of muscle atrophy in the neck.

## 1. Introduction

The unique musculoskeletal and spinal pain issues of astronauts under microgravity conditions have been clinically confirmed [1,2]. Intervertebral disc herniation is one of the most common symptoms observed in astronauts in a microgravity environment [1,2]. Previous studies have shown that approximately 10% of American astronauts experience intervertebral disc herniation after spaceflight, predominantly in the cervical and lumbar regions [1,2]. Astronauts typically report subjective experiences of lower back and neck pain, which, in more severe cases, may lead to radiculopathy, ultimately causing disability [3]. A comparative study found that the incidences of cervical and lumbar disc herniation in astronauts returning from space were 21.4 times and 4.3 times higher, respectively, than those of the matched controls on Earth [2]. Similarly, NASA’s studies have found that the risk of cervical disc herniation in astronauts is 35.9 times that of the control group, and the risk of cervical disc herniation is 2.8 times higher than that of lumbar disc herniation [4].

Regarding the mechanisms of lumbar disc herniation under microgravity conditions, Belavy et al. suggested that the unloading of the lumbar joint capsule in microgravity may lead to disc swelling, which could be the primary mechanism for lumbar disc herniation [2]. Later, Wu et al.’s numerical study further identified increased lumbar disc volume and increased disc hydration as key pathological mechanisms [5]. Therefore, current research on intervertebral disc herniation mechanisms under microgravity mainly focuses on the lumbar intervertebral disc, and its mechanisms are relatively well understood. However, there are limited data on the mechanisms of cervical disc herniation and no public data from astronauts on cervical disc changes during spaceflight [6,7]. Additionally, there is a lack of data on the adaptive changes in cervical neuromuscular function under microgravity conditions [2]. Although it is known that during spaceflight, the spine elongates by 4–7 cm due to disc swelling, which is one of the pathological mechanisms of disc herniation [8], it remains unclear whether similar effects occur in the cervical spine, as well as how these changes impact adjacent intervertebral discs. Overall, the incidence of cervical disc herniation in microgravity is several times higher than that of lumbar disc herniation. However, due to the unique geometric characteristics of cervical intervertebral discs and the challenges associated with collecting data on cervical muscle activity, there has been limited foundational research into the underlying pathological mechanisms [2].

Currently, musculoskeletal models are widely employed in scenarios where the direct measurement of specific parameters or indicators is challenging, particularly in cases where human testing is not feasible due to ethical concerns or restrictive experimental conditions. The Anybody Modeling System (AnyBody Technology A/S, Aalborg, Denmark), as one of the musculoskeletal modeling simulation software, can simulate various muscles, ligaments, and bones during human movement through mathematical modeling techniques and calculate dynamic indicators using inverse dynamics methods [9,10]. This method has become a crucial tool for investigating human motion, ergonomics, and biomechanics in extreme environments, with the validity of its results having been extensively validated [11,12,13,14,15]. Therefore, using a musculoskeletal model to simulate the mechanical properties of cervical spine segments appears to be an effective approach to delving into this pathological mechanism.

**Figure 1:**
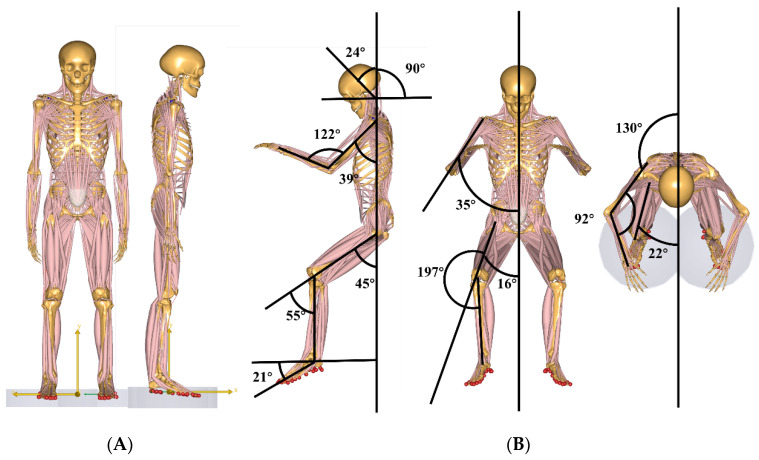
Simulated Postures (**A**) neutral body posture in microgravity (NBP 0G) and standing posture in microgravity (SM 0G); (**B**) neutral body posture in microgravity (NBP 0G).

The neutral body posture (NBP) in space significantly differs from that on Earth. In a microgravity environment, the NBP is characterized by a relaxed posture, whereas on Earth, the NBP is typically a relaxed standing posture, as illustrated in Figure 1 [16,17,18,19]. Compared to the NBP on Earth, the NBP in microgravity causes the neck to tilt forward (flexed) by about 24° [5]. This behavior results in a reduction in the natural lordosis of the cervical spine, thereby affecting its alignment in the sagittal plane [20]. During spaceflight, we speculate that the cervical spine is subjected to traction forces, which further influence its posture. Combined with the load loss across the intervertebral discs, this undoubtedly leads to changes in the microstructure of the discs. These changes may include alterations in the internal pressure distribution of the intervertebral disc, stress concentration in the annulus fibrosus (AF), and impaired exchange of water and nutrients in the nucleus pulposus (NP), thereby increasing the risk of cervical disc herniation [21]. Moreover, during space operations, astronauts mostly adopt the floating posture, which is the most commonly used posture in space. From an anatomical perspective, and given the frequency of posture adoption, this position may serve as a contributing factor to cervical disc herniation. It remains unclear how the mechanical load on cervical spine segments in this posture is affected under microgravity conditions. Whether the biomechanical load on cervical spine segments in microgravity differs from that in the Earth’s gravitational environment under the NBP is still an open question. Furthermore, whether these differences may be related to the formation of cervical disc herniation is also a topic worth further exploration.

This study aimed to utilize the Anybody musculoskeletal model to investigate the effects of various NBPs on the cervical spine and its associated muscle mechanical properties under both gravitational and microgravity conditions. This study is of significant importance for understanding the biomechanical mechanisms underlying cervical disc herniation related to microgravity. Leveraging this computational model, the research could contribute to the development of strategies for treating acute cervical radiculopathy in microgravity, as well as offer valuable insights for the design and implementation of spinal countermeasures in future microgravity environments. Given the reduction in external forces acting on the human body in microgravity, we hypothesized that the compressive and shear forces on the C2–C7 cervical discs in microgravity would decrease compared to those in a gravitational environment.

## 2. Methods

### 2.1. Musculoskeletal Model

**Figure 2:**
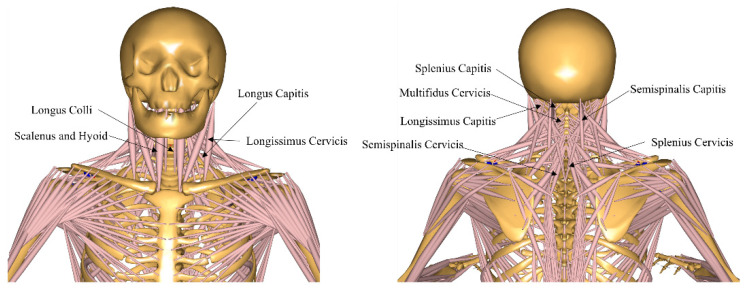
Major muscular groups of the cervical spine region in the anybody musculoskeletal model.

To investigate the effects of different gravitational environments on the biomechanical changes in the cervical spine, this study utilized a full-body musculoskeletal model from the AMMR database developed by the Anybody Modeling System. The model is based on the anatomical structure and body dimensions of an adult male (height: 1.74 m, body mass: 72 kg; 720 N). The spinal model used in this study has been validated through previous cadaveric and in vivo loading tests, as well as using intragroup correlation coefficients of electromyographic envelopes, ensuring its high accuracy [13,22,23,24]. Furthermore, a previous simulation study on musculoskeletal models in microgravity environments also discussed the validity of the results of the present model [5]. The discussion indicates that the model demonstrates high validity in terms of the ligament and intervertebral disc height metrics. The cervical spine model in this musculoskeletal model includes seven cervical vertebrae, seven intervertebral discs, three cervical ligaments (anterior longitudinal ligament [ALL], posterior longitudinal ligament [PLL], and ligaments flava [LF]), as well as the following eleven major cervical muscles: scalenus and hyoid muscles; longus colli; longus capitis; splenius capitis; splenius cervicis; semispinalis capitis; semispinalis cervicis; longissimus capitis; longissimus cervicis; and multifidus cervicis (Figure 2).

In terms of mechanical behavior, the cervical spine region muscles are modeled as ‘full’, the muscle type is defined as ‘simple’, and the cervical vertebrae are represented as rigid bodies. The constitutive relationship of the intervertebral discs is modeled using a six-degree-of-freedom rotation–translation coupled joint [25], which expresses its mechanical characteristics through the following two key coupling relationships: the rotational deformation–displacement response mechanism driven by the conservation of angular momentum equation, and the translational deformation–stress constitutive relationship derived from the linear momentum theorem. This dual-coupling modeling approach allows the intervertebral discs’ nonlinear mechanical response characteristics to be represented in a three-dimensional space. The ligaments are modeled as a piecewise linear model, where stiffness depends on the strain, and the values are taken from previous cadaveric studies. The stiffness values can be found in the research by Yoganandan et al. [26] and are briefly listed in Table 1. For muscles, the maximum muscle strength is assumed to be related to the functional cross-sectional area [22].

To calculate the water content of the nucleus pulposus (NP) and annulus fibrosus (AF) of the cervical intervertebral disc under swelling effects during microgravity unloading, the triphasic theory model for cartilage swelling and deformation behavior was employed. This model divides the cartilage into three phases—Solid Phase, Fluid Phase, and Swelling Phase—to explain the physical response of cartilage under varying loads, permeability, and deformation conditions.

Step 1: Solving for the deformation-dependent swelling pressure $F_{s}$.

(1) $$F_{s}=RT\left(\sqrt{(c_{0}^{F}\frac{\phi_{0}^{w}}{\phi_{0}^{w}+{\left(h/h_{0}\right)}^{3}-1})+4c^{{*}^{2}}}-2c^{*}\right)$$

where R is the universal gas constant $(8.3144\;\left.{JK}^{-1}mol\right)$, T is the temperature in Kelvin (310.15 K), and h refers to the height of the intervertebral disc after deformation due to external forces (such as load or the swelling effect after load removal). This height reflects the swelling or compression state of the disc, which typically varies with changes in the disc’s water content and swelling pressure. $h_{0}$ represents the reference height of the intervertebral disc, which corresponds to the natural height of the disc under normal gravity conditions, reflecting the initial volume or morphology of the disc (Table 2). c* is the concentration of Na^+^ and Cl^−^ in the surrounding environment of the intervertebral disc (150 mM). The term $c_{0}^{F}\frac{\phi_{0}^{w}}{\phi_{0}^{w}+{\left(h/h_{0}\right)}^{3}-1}$ represents the fixed charge density (FCD) inside the disc, which depends on the disc’s deformation and is primarily derived from glycosaminoglycans (such as chondroitin sulfate and keratan sulfate). These fixed charges attract water molecules, and therefore they play a significant role in the swelling behavior of the intervertebral disc [27].

Step 2: Solving for water content $\phi^{w}$ of the deformed disc.

(2) $$\phi^{w}=\frac{\phi_{0}^{w}+{\left(h/h_{0}\right)}^{3}-1}{{\left(h/h_{0}\right)}^{3}}$$

where $\phi_{0}^{w}$ is the water content of the intervertebral disc in the reference state (specific value provided in Table 2).

Step 3: Solving for the water content in NP and AF.

(3) $$\phi^{w}=0.4\phi_{NP}^{w}+0.6\phi_{AF}^{w}$$

In this study, based on the experimental data [28], it was assumed that the average FCD in the AF of a healthy intervertebral disc would be 80% of that in the NP. Additionally, drawing on the cadaveric experimental data from Pooni et al. [29], it was assumed that the cross-sectional area of the NP would be 40% of the total cross-sectional area of the intervertebral disc.

#### Research Posture

This study conducted a quantitative analysis of three postures, including the neutral body posture in microgravity (NBP 0G), the neutral body posture in a gravity environment (NBP 1G), and the standing posture in microgravity (SM 0G).

The static kinematic parameters for the NBP 1G and SM 0G postures were derived from the original kinematics of the Standing Model in the AMMR database of the Anybody Modeling System. Specifically, the parameters were sternoclavicular joint protraction 23°, sternoclavicular joint elevation 11.5°, shoulder joint flexion 8°, shoulder abduction 10°, elbow joint flexion 8°, elbow joint pronation −20°, hip flexion −6°, hip abduction 5°, with all other joint angles set to 0° (see Figure 1A).

NBP 0G was based on the kinematic data of NBP collected during a six-month space flight aboard the Russian space station, as reported by Andreoni et al. [18].The specific values were as follows: Compared to NBP 1G, the neck flexion was 24°, with a 15° reduction in the line of sight. Shoulder flexion was 39°, shoulder abduction was 35°, elbow flexion was 77°, and elbow pronation was 60°. Hip flexion was 55°, hip abduction was 16°, and hip external rotation was 17°. Knee flexion was 55°, and ankle plantarflexion was 21°. All other joint angles were set to 0° [5]. In the Anybody Modeling System, these kinematic modifications were implemented by adjusting the gravity parameters to 0G in the Standing Model.Main.any file and modifying the joint kinematics in the Posture folder of the Model\Mannequin.any file.

### 2.2. Research Variables

Previous studies have shown that the primary variables influenced by microgravity, which affect cervical intervertebral disc degeneration and cervical disc herniation, include disc loading, the water content of the NP and AF, structural changes in the disc, ligament forces, and joint muscle forces [5]. Therefore, the research variables included in this study were the intervertebral disc forces (compression and shear forces at the C2–T1 joints), the water content of the NP and AF, changes in the disc height, changes in the disc cross-sectional area, changes in the disc volume, muscle forces of the cervical joint muscle groups (Scalenus and Hyoid; Longus Colli; Longus Capitis; Splenius Capitis; Splenius Cervicis; Semispinalis Capitis; Semispinalis Cervicis; Longissimus Capitis; Longissimus Cervicis; Multifidus Cervicis), and cervical joint ligament forces (C2–C5, C5–T1), including the ALL, PLL, and LF.

## 3. Results

### 3.1. Cervical Disc Compressive Force and Shear Force

Under NBP 1G, the compressive forces for the C2–C3, C3–C4, C4–C5, C5–C6, C6–C7, and C7–T1 discs were 91.83 N, 101.69 N, 110.37 N, 121.15 N, 144.87 N, and 178.07 N, respectively. The shear forces for these discs were −13.27 N, −15.44 N, −11.29 N, −12.61 N, −10.67 N, and −7.9 N, respectively (Table S1).

Under SM 0G, the compressive forces for the six discs were 22.11 N, 24.98 N, 27.17 N, 28.92 N, 33.37 N, and 39.37 N, respectively. The shear forces for these discs were −4.3 N, −4.43 N, −1.53 N, −1.06 N, 0.09 N, and 0.29 N, respectively. The shear forces at C6–C7 and C7–T1 became positive.

**Figure 3:**
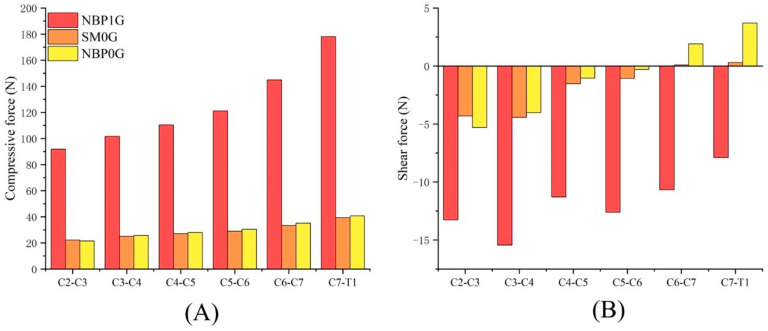
Changes in cervical disc mechanics under three postures. (**A**) Compressive force; (**B**) Shear force. The direction of the shear force is based on the sagittal plane, where the positive direction (+) points behind the sagittal plane and the negative direction (−) points to the front of the sagittal plane.

Under NBP 0G, the compressive forces for the six discs were 21.5 N, 25.77 N, 27.9 N, 30.5 N, 35.2 N, and 40.7 N, respectively. The shear forces for these discs were −5.3 N, −4.02 N, −1.05 N, −0.3 N, 1.9 N, and 3.7 N, respectively. The direction of the shear forces changed at C6–C7 and C7–T1 (Figure 3).

#### 3.1.1. NBP 1G vs. SM 0G

Compared to NBP 1G, the cervical disc compressive force under SM 0G decreased, while the shear force increased. The compressive forces for the C2–C3, C3–C4, C4–C5, C5–C6, C6–C7, and C7–T1 discs decreased by 75.9%, 75.4%, 75.4%, 76.1%, 77.0%, and 77.9%, respectively. The shear forces increased by 8.97 N, 11.01 N, 9.76 N, 11.55 N, 10.76 N, and 8.19 N, respectively.

#### 3.1.2. NBP 1G vs. NBP 0G

Compared to NBP 1G, the cervical disc compressive force under NBP 0G decreased, while the shear force increased. The compressive forces for the C2–C3, C3–C4, C4–C5, C5–C6, C6–C7, and C7–T1 discs decreased by 76.6%, 74.7%, 74.7%, 74.8%, 75.7%, and 77.1%, respectively. The shear forces increased by 7.97 N, 11.42 N, 10.24 N, 12.31 N, 12.57 N, and 11.6 N, respectively.

#### 3.1.3. NBP 0G vs. SM 0G

Under NBP 0G, the cervical disc compressive forces at C3–C4, C4–C5, C5–C6, C6–C7, and C7–T1 were higher than those under SM 0G, at 103.2%, 102.7%, 105.5%, 105.5%, and 103.4%, respectively. The compressive force at C2–C3 under NBP 0G was 2.8% lower than that under SM 0G.

In terms of shear forces, NBP 0G showed higher shear forces at C3–C4, C4–C5, and C5–C6 compared to SM 0G, with increases of 0.41 N, 0.48 N, and 0.76 N, respectively. At C6–C7 and C7–T1, the shear forces under NBP 0G were significantly higher than those under SM 0G, reaching 2011.1% and 1175.9% of the values seen under SM 0G, respectively.

### 3.2. Cervical Disc Geometric

Under NBP 1G, the disc heights for the C2–C3, C3–C4, C4–C5, C5–C6, C6–C7, and C7–T1 levels were 3.1 mm, 3.51 mm, 4.12 mm, 5.01 mm, 4.2 mm, and 3.7 mm, respectively. The cross-sectional areas were 380.00 mm^2^, 420.00 mm^2^, 490.00 mm^2^, 530.00 mm^2^, 540.00 mm^2^, and 370.00 mm^2^, respectively. The disc volumes were 1178 mm^3^, 1474.2 mm^3^, 2018.8 mm^3^, 2655.3 mm^3^, 2268 mm^3^, and 1369 mm^3^, respectively (Table S2).

Under SM 0G, the disc heights for the C2–C3, C3–C4, C4–C5, C5–C6, C6–C7, and C7–T1 levels were 3.13 mm, 3.56 mm, 4.2 mm, 5.2 mm, 4.27 mm, and 3.75 mm, respectively. The cross-sectional areas were 390.00 mm^2^, 440.00 mm^2^, 512.00 mm^2^, 542.00 mm^2^, 570.00 mm^2^, and 390.00 mm^2^, respectively. The disc volumes were 1220.7 mm^3^, 1566.4 mm^3^, 2150.4 mm^3^, 2814.4 mm^3^, 2433.9 mm^3^, and 1462.5 mm^3^, respectively.

**Figure 4:**
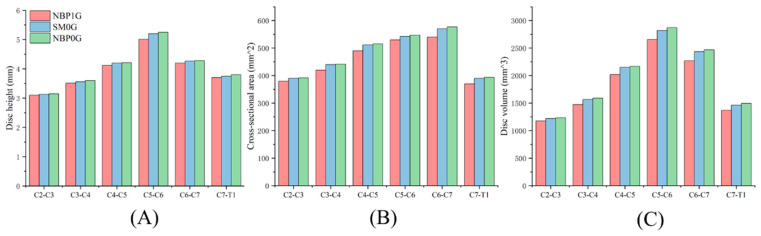
Geometric changes of cervical discs under three postures. (**A**) Disc height. (**B**) Disc cross-sectional area. (**C**) Disc volume.

Under NBP 0G, the disc heights for the C2–C3, C3–C4, C4–C5, C5–C6, C6–C7, and C7–T1 levels were 3.15 mm, 3.6 mm, 4.21 mm, 5.25 mm, 4.28 mm, and 3.8 mm, respectively. The cross-sectional areas were 392.00 mm^2^, 442.00 mm^2^, 515.00 mm^2^, 547.00 mm^2^, 577.00 mm^2^, and 394.00 mm^2^, respectively. The disc volumes were 1234.8 mm^3^, 1591.2 mm^3^, 2168.15 mm^3^, 2871.75 mm^3^, 2469.56 mm^3^, and 1497.2 mm^3^, respectively (Figure 4).

#### 3.2.1. NBP 1G vs. SM 0G

In SM 0G, the cervical disc geometry was larger than in NBP 1G. The disc heights for C2–C3, C3–C4, C4–C5, C5–C6, C6–C7, and C7–T1 increased by 1.0%, 1.4%, 1.9%, 3.8%, 1.7%, and 1.4%, respectively, compared to NBP 1G. The cross-sectional areas increased by 2.6%, 4.8%, 4.5%, 2.3%, 5.6%, and 5.4%, respectively. The disc volumes increased by 3.6%, 6.3%, 6.5%, 6.1%, 7.3%, and 6.8%, respectively.

#### 3.2.2. NBP 1G vs. NBP 0G

In NBP 0G, the cervical disc geometry was larger than in NBP 1G. The disc heights for C2–C3, C3–C4, C4–C5, C5–C6, C6–C7, and C7–T1 increased by 1.6%, 2.6%, 2.2%, 4.8%, 1.9%, and 2.7%, respectively, compared to NBP 1G. The cross-sectional areas increased by 3.2%, 5.2%, 5.1%, 3.2%, 6.9%, and 6.5%, respectively. The disc volumes increased by 4.8%, 7.9%, 7.4%, 8.2%, 8.9%, and 9.4%, respectively.

#### 3.2.3. NBP 0G vs. SM 0G

Compared to NBP 0G, the cervical disc geometry under SM 0G was smaller. The disc heights for C2–C3, C3–C4, C4–C5, C5–C6, C6–C7, and C7–T1 decreased by 0.4%, 4.1%, 5.1%, 1.4%, 8.3%, and 6.5%, respectively. The cross-sectional areas decreased by 1.1%, 1.8%, 3.3%, 4.4%, 1.4%, and 1.7%, respectively. The disc volumes decreased by 1.5%, 6.0%, 1.9%, 5.8%, 9.9%, and 8.3%, respectively.

### 3.3. Cervical Joint Muscle Force

#### 3.3.1. NBP 1G vs. SM 0G

In NBP 1G, the muscle forces of the Scalenus and Hyoid, Longus Colli, Semispinalis Capitis, Semispinalis Cervicis, Longissimus Cervicis, and Multifidus Cervicis were greater than in SM 0G. The force increase compared to SM 0G was 8.41 N, 2.87 N, 2.78 N, 12.75 N, 0.69 N, and 11.26 N, respectively. However, the muscle forces of the Longus Capitis, Splenius Capitis, Splenius Cervicis, and Longissimus Capitis in NBP 1G were smaller than those in SM 0G, with decreases of −1.41369 × 10^−5^, −6.2668 × 10^−8^ N, −1.40414 × 10^−6^ N, and −1.00747 × 10^−5^ N, respectively (Table 3).

#### 3.3.2. NBP 1G vs. NBP 0G

In NBP 1G, the muscle forces of the Scalenus and Hyoid, Longus Colli, Splenius Capitis, Semispinalis Capitis, Semispinalis Cervicis, Longissimus Cervicis, and Multifidus Cervicis were greater than in NBP 0G. The force increase compared to NBP 0G was 8.41 N, 2.87 N, 9.44 × 10^−14^ N, 2.78 N, 12.75 N, 0.69 N, and 11.26 N, respectively. However, the muscle forces of the Longus Capitis, Splenius Cervicis, and Longissimus Capitis in NBP 1G were smaller than those in NBP 0G, with decreases of −1.85277 × 10^−5^ N, 9.44293 × 10^−14^ N, −4.19436 × 10^−7^ N, and −1.17697 × 10^−5^ N, respectively.

#### 3.3.3. NBP 0G vs. SM 0G

In NBP 0G, the muscle forces of the Scalenus and Hyoid, Longus Colli, Longus Capitis, Semispinalis Capitis, Longissimus Capitis, Longissimus Cervicis, and Multifidus Cervicis were greater than in SM 0G. The force increase compared to SM 0G was 1.90005 × 10^−6^ N, 2.04155 × 10^−5^ N, 4.39074 × 10^−6^ N, 4.49806 × 10^−6^ N, 1.69498 × 10^−6^ N, 5.59013 × 10^−7^ N, and 7.26436 × 10^−6^ N, respectively. However, the muscle forces of the Splenius Capitis, Splenius Cervicis, and Semispinalis Cervicis in NBP 0G were smaller than those in SM 0G, with decreases of −6.26681 × 10^−8^ N, −9.84702 × 10^−7^ N, and −3.33825 × 10^−7^ N, respectively.

### 3.4. Intervertebral Disc Water Content

In NBP 1G, the water content of the NP for each intervertebral disc was as follows: C2C3: 80.0%, C3C4: 82.0%, C4C5: 81.0%, C5C6: 83.0%, C6C7: 82.0%, C7T1: 81.0%. The water content of the AF was as follows: C2C3: 75.0%, C3C4: 76.0%, C4C5: 76.0%, C5C6: 78.0%, C6C7: 77.0%, C7T1: 76.0% (Table S3).

In SM 0G, the water content of the NP for each intervertebral disc was as follows: C2C3: 85.0%, C3C4: 86.6%, C4C5: 86.8%, C5C6: 85.0%, C6C7: 83.0%, C7T1: 83.5%. The water content of the AF was as follows: C2C3: 77.0%, C3C4: 78.8%, C4C5: 78.9%, C5C6: 78.8%, C6C7: 78.0%, C7T1: 76.2%.

**Figure 5:**
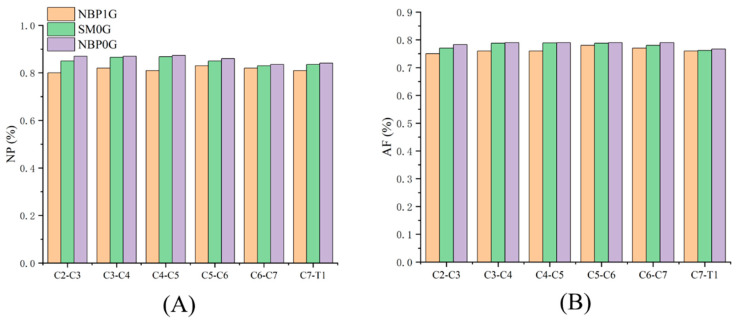
Intervertebral disc water content under three postures. (**A**) NP water content; (**B**) AF water content.

In NBP 0G, the water content of the NP for each intervertebral disc was as follows: C2C3: 87.0%, C3C4: 87.0%, C4C5: 87.4%, C5C6: 86.0%, C6C7: 83.5%, C7T1: 84.1%. The water content of the AF was as follows: C2C3: 78.3%, C3C4: 79.0%, C4C5: 79.0%, C5C6: 79.0%, C6C7: 79.0%, C7T1: 76.7% (Figure 5).

#### 3.4.1. NBP 1G vs. SM 0G

Compared to NBP 1G, the intervertebral disc water content in SM 0G increased. The water content of the NP at C2C3, C3C4, C4C5, C5C6, C6C7, and C7T1 increased by 6.2%, 5.6%, 7.2%, 2.4%, 1.2%, and 3.1%, respectively. The water content of the AF increased by 2.7%, 3.7%, 3.8%, 1.0%, 1.3%, and 0.3%, respectively.

#### 3.4.2. NBP 1G vs. NBP 0G

Compared to NBP 1G, the intervertebral disc water content in NBP 0G increased. The water content of the NP at C2C3, C3C4, C4C5, C5C6, C6C7, and C7T1 increased by 8.8%, 6.1%, 7.9%, 3.6%, 1.8%, and 3.8%, respectively. The water content of the AF increased by 4.4%, 3.9%, 3.9%, 1.3%, 2.6%, and 0.9%, respectively.

#### 3.4.3. NBP 0G vs. SM 0G

Compared to SM 0G, the intervertebral disc water content in NBP 0G increased. The water content of the NP at C2C3, C3C4, C4C5, C5C6, C6C7, and C7T1 increased by 2.4%, 0.5%, 0.7%, 1.2%, 0.6%, and 0.7%, respectively. The water content of the AF increased by 1.7%, 0.3%, 0.1%, 0.3%, 1.3%, and 0.7%, respectively.

### 3.5. Ligament Forces

**Figure 6:**
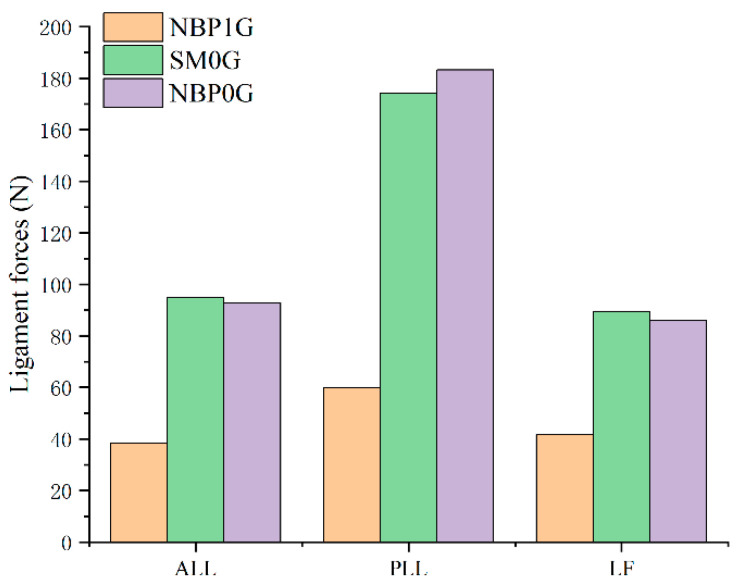
Ligament forces under three postures.

In NBP 1G, the forces on the ALL, PLL, and LF were 38.46 N, 59.95 N, and 41.86 N, respectively. In SM 0G, the forces on the ALL, PLL, and LF were 95.02 N, 174.21 N, and 89.37 N, respectively. In NBP 0G, the forces on the ALL, PLL, and LF were 92.76 N, 183.26 N, and 85.97 N, respectively (Figure 6).

In SM 0G, the forces on ALL, PLL, and LF were greater than those in NBP 1G. Compared to NBP 1G, the forces on the corresponding ligaments in SM 0G increased by 147.1%, 190.6%, and 113.5%, respectively (Table S4).

In NBP 0G, the forces on the ALL, PLL, and LF were greater than those in NBP 1G. Compared to NBP 1G, the forces on the corresponding ligaments in NBP 0G increased by 141.2%, 205.7%, and 105.4%, respectively.

In SM 0G, the forces on the ALL and LF were greater than those in NBP 0G. Compared to NBP 0G, the forces on the corresponding ligaments in SM 0G increased by 2.4% and 3.9%, respectively. However, the force on the PLL in SM 0G was lower than that in NBP 0G, with a decrease of 4.9%.

## 4. Discussion

Due to the uniqueness of the microgravity environment, there is currently a lack of fundamental research on the pathological mechanisms of cervical disc herniation related to microgravity. This study employed musculoskeletal modeling methods to investigate the effects of three different neutral postures (NBP1G, SM0G, NBP0G) on the mechanical properties of the C2–T1 intervertebral discs and their associated muscles and ligaments in both gravitational and microgravity environments. The results of this study were consistent with our initial hypothesis, which stated that the compressive forces on the cervical intervertebral discs in microgravity were lower compared to those in a gravitational environment. Additionally, the study revealed that the shear forces on the intervertebral discs in microgravity, compared to the gravitational conditions, exhibited a decreasing trend in the negative direction, and the direction of these forces changed at the C6–C7 and C7–T1 disc levels. These findings partially support our earlier hypotheses. Moreover, our results suggest that, compared to the NBP1G posture, conditions in SM0G and NBP0G lead to increases in the intervertebral disc water content, disc height, cross-sectional area, volume, and ligament forces, while the resultant muscle forces of the cervical musculature decreased. However, for certain muscles, the force values under SM0G and NBP0G were greater than those under NBP1G.

The geometric and water content simulation results for the intervertebral discs in this study were within the reasonable ranges. A study using dry immersion to simulate a microgravity environment indicated that the NP water content in the C2–C3 and C7–T1 intervertebral discs of the human body in microgravity would increase by 4.58% and 4.70%, respectively, compared to the gravitational conditions [30]. In the present study, the NP water content in the C2–C3 and C7–T1 discs under SM0G increased by 6.2% and 3.1%, respectively, compared to NBP1G, while the NP water content in the same discs under NBP0G increased by 8.8% and 3.8% compared to NBP1G. A previous study measured the height of 29 crew members before and during space flight. The results showed that, in microgravity, the height of these crew members increased by more than 6% [31]. Based on this measurement, the study further inferred that the increase in vertebral disc height in microgravity compared to ground conditions ranged from 1.05% to 3% [5,31]. In our study, compared to the NBP1G condition, the increase in cervical intervertebral disc height under SM0G ranged from 1% to 3.8%, while the increase under NBP0G ranged from 1.6% to 4.8%. Most of the intervertebral discs in this study showed an increase in height within the range observed in prior studies, which is in close agreement with the actual results.

Previous studies have shown that the axial force unloading of intervertebral discs and the loss of cervical lordosis curvature in a microgravity environment lead to disc expansion, resulting in spinal elongation [32,33]. Our findings align with these previous observations. We observed that, compared to the NBP1G posture, the axial load on each intervertebral disc under the NBP0G condition was reduced by an average of 76.6%, while the disc height increased by an average of 2.63%. An ex vivo study suggested that the increase in disc height caused by unloading might lead to the protrusion of the NP at the posterior–lateral corner, which can put pressure on the spinal cord or nerve roots, potentially causing pain and nerve function damage [34]. Additionally, a NASA Case Report identified NP protrusion at the C6–C7 level in astronauts exposed to microgravity, with the patient subsequently developing unilateral C7 sensory-motor radiculopathy [1]. Thus, the increase in intervertebral disc height caused by unloading may contribute to the development of cervical intervertebral disc herniation and pain [4]. We also found that under the NBP0G condition, the direction of the shear force on the C6–C7 and C7–T1 discs was opposite to that observed under the NBP1G condition. This may result from posterior–lateral compression of the NP, which altered the overall force distribution across the disc. The change in shear force direction at these disc levels corresponded with the location of NP protrusion observed in a previous case report by Scheuring et al. [1]. This result suggests that the change in shear force direction in the C6–C7 and C7–T1 discs may cause the nucleus pulposus to shift off-center and be displaced posteriorly under external forces. Therefore, in addition to the increase in disc height, the alteration of shear force direction in the intervertebral disc may also be one of the factors contributing to cervical disc herniation and pain.

In a microgravity environment, the unloading of the disc’s compressive force not only causes changes in the external geometry of the intervertebral disc but can also influence the synthesis of the disc’s matrix (glycosaminoglycans, GAGs) [35]. Glycosaminoglycans are key chemical components of the intervertebral disc matrix, and alterations in GAG synthesis may increase the risk of disc degeneration [36]. Previous studies have shown that as compressive forces on the intervertebral disc decrease, the rate of GAG synthesis significantly declines [35,36,37]. One study found that under conditions where the axial load on the NP was 300% higher than the original load, the GAG synthesis rate decreased by 74%, and under conditions where the axial load was 90% lower than the original, GAG synthesis decreased by 80% [38]. In this study, compared to NBP1G, the compressive force on each cervical intervertebral disc under NBP0G decreased by an average of 76.6%, leading us to hypothesize that the GAG synthesis rate under NBP0G would be significantly reduced. Additionally, the increase in intervertebral disc volume in a microgravity environment could result in decreased transportation rates of GAGs within the disc, which is an important phenomenon to address [39,40]. The intervertebral disc is an avascular structure, and its nutrients are absorbed at the outer edges of the AF and then transported by diffusion to the central NP. However, an increase in diffusion distance would slow the diffusion rate of GAGs [39,40]. Therefore, these two factors—the reduced synthesis rate and slower transportation of GAGs—could contribute to a decline in disc health and potentially lead to degenerative disc diseases [41].

The ligament forces under the NBP0G condition were greater than those under the NBP1G posture. This can be attributed to the swelling of the intervertebral discs in microgravity, which increases the tension on the spinal ligaments, leading to greater ligament length and force [42,43]. Furthermore, the significant cervical flexion posture in NBP0G results in higher tension on the PLL compared to the SM0G condition. This observation aligns with the previous studies which found that cervical flexion increases ligament tension [42,43]. Previous research has demonstrated that the increase in ligament tension contributes to more compression on the cervical intervertebral discs, especially when the spine is in a flexed position, which may elevate the risk of intervertebral disc herniation during space missions [42]. A prior study suggested that increased ligament tension in microgravity could also contribute to potential ligament damage in structures such as the ligamentum flavum (LF), interspinous ligaments, and supraspinous ligaments [5]. A comparison of our results with the failure characteristics of cervical ligaments reported by Bass et al. [44] showed that the ligament tension under NBP0G has not yet reached the failure thresholds. For example, the forces for the ALL under NBP0G were 92.76 N, compared to the failure threshold of 494 N; for the PLL, NBP0G had 183.26 N, compared to the failure threshold of 462.9 N; and for the LF, NBP0G had 85.97 N, compared to the failure threshold of 315.8 N. This suggests that, although ligament forces are elevated in the NBP0G posture, they remain well below the thresholds for ligament failure.

In contrast to the NBP1G posture, the total muscle force in the cervical spine joints under NBP0G decreased. This reduction in muscle force may contribute to muscle atrophy, a phenomenon frequently observed in astronauts who have spent extended periods in microgravity and then return to Earth. Muscle weakness and neuromuscular dysfunction are known factors that may contribute to an increased risk of intervertebral disc degeneration [45]. The reduction in muscle force, particularly in the neck and upper body, could also compromise spinal stability and increase the risk of disc-related complications in a microgravity environment.

### Limitations and Future Directions

The limitations we acknowledge will need to be addressed in future research. For one, the sample population in this study was limited to a male adult (height: 1.74m, mass: 72 kg). While the model used in this study is one of the few that has been validated for spinal forces and moments, the single sample introduces the potential for individual variation that could affect the generalizability of the findings. In particular, the anatomical differences between astronauts (or between individuals in general) were not fully accounted for in this study. Future research should incorporate a more diverse sample, including various body types, genders, and anatomies, to more accurately represent the broader population.

Due to the limited data available on intervertebral discs in microgravity environments, the mechanical parameters of intervertebral discs in this study were only partially validated. Furthermore, as there are currently no relevant cadaveric studies to validate cervical ligament forces, this study has certain limitations in this regard. The musculoskeletal model employed also cannot simulate the long-term adaptive changes in ligaments or muscles. Consequently, the potential adaptive changes in the ligaments under prolonged microgravity exposure were not accounted for in this study.

Additional studies are required to refine the models and expand their scope to more precisely represent the physiological changes occurring in microgravity.

## 5. Conclusions

Compared to the gravitational environment, in the microgravity environment with the NBP0G posture, the intervertebral disc compressive force and shear force in the same direction were reduced, while the shear force direction in the C6C7 and C7T1 segments changed. Additionally, the intervertebral disc’s geometric dimensions increased, the total muscle force in the cervical spine decreased, and the ligament tension in the cervical joints increased. These changes may increase the likelihood of cervical disc herniation and related diseases, as well as the risk of cervical muscle atrophy.

## Figures and Tables

**Table 1:** Stiffness and Strain Values of Cervical Intervertebral Disc Ligaments.

	Ligament Stiffness [N/mm]	Strain [%]
**ALL**	16	25.8–35.8
	17.9	29.54–41.26
**PLL**	25.4	14.99–21.41
	23	25.33–42.87
**LF**	25	64.1–89.9
	21.6	101.5–75.3

**Table 2:** Disc Cross-Section, Water Content Proportions, and Heights in the Model.

	A0 [mm^2^]	$\phi^{w}$		h0 [mm]
		NP	AF	
**C2–C3**	380	0.80	0.75	3.1
**C3–C4**	420			3.51
**C4–C5**	490			4.12
**C5–C6**	530			5.01
**C6–C7**	540			4.2
**C7–T1**	370			3.7

**Table 3:** Cervical region muscle forces under three postures.

Muscle (N)	NBP1G	SM0G	NBP0G
Scalenus And Hyoid	8.41	9.55 × 10^−6^	1.14 × 10^−5^
Longus Colli	2.87	2.92 × 10^−5^	4.96 × 10^−5^
Longus Capitis	9.94 × 10^−13^	1.41 × 10^−5^	1.85 × 10^−5^
Splenius Capitis	9.44 × 10^−14^	6.27 × 10^−8^	0
Splenius Cervicis	2.78 × 10^−13^	1.40 × 10^−6^	4.19 × 10^−7^
Semispinalis Capitis	2.78	1.09 × 10^−5^	1.54 × 10^−5^
Semispinalis Cervicis	12.75	4.58 × 10^−6^	4.25 × 10^−6^
Longissimus Capitis	3.96 × 10^−12^	1.01 × 10^−5^	1.18 × 10^−5^
Longissimus Cervicis	0.69	1.92 × 10^−5^	1.98 × 10^−5^
Multifidus Cervicis	11.3	1.04 × 10^−5^	1.77 × 10^−5^

## Data Availability

Data will be made available on request.

## Supplementary Materials

The following supporting information can be downloaded at: https://www.mdpi.com/article/10.3390/life15030447/s1, Table S1: Changes in cervical disc mechanics under three postures; Table S2: Intervertebral disc water content under three postures; Table S3: Geometric changes of cervical discs under three postures; Table S4 Ligament forces under three postures.
