# Supplementary material for: Effects of Neutral Postures on Mechanical Properties of Cervical Spine Under Different Gravitational Environments: A Musculoskeletal Model Study
Source: Life (Basel). 2025 Mar 12;15(3):447. doi: 10.3390/life15030447 (PMC11944035; doi:10.3390/life15030447)
Supplement: Supplementary file 1 [file life-15-00447-s001.zip › life-3503710-supplementary.pdf]

Table S1. Changes in cervical disc mechanics under three postures.

Variable	Compressive force	Compressive force	Compressive force	Shear force	Shear force	Shear force
Unit	N	N	N	N	N	N
Posture	NBP1G	SM0G	NBP0G	NBP1G	SM0G	NBP0G
C2-C3	91.83	22.11	21.5	-13.27	-4.3	-5.3
C3-C4	101.69	24.98	25.77	-15.44	-4.43	-4.02
C4-C5	110.37	27.17	27.9	-11.29	-1.53	-1.05
C5-C6	121.15	28.92	30.5	-12.61	-1.06	-0.3
C6-C7	144.87	33.37	35.2	-10.67	0.09	1.9
C7-T1	178.07	39.37	40.7	-7.9	0.29	3.7

Table S2. Intervertebral disc water content under three postures.

Variable	NP	NP	NP	AF	AF	AF
Unit	%	%	%	%	%	%
Posture	NBP1G	SM0G	NBP0G	NBP1G	SM0G	NBP0G
C2-C3	0.8	0.85	0.87	0.75	0.77	0.783
C3-C4	0.82	0.866	0.87	0.76	0.788	0.79
C4-C5	0.81	0.868	0.874	0.76	0.789	0.79
C5-C6	0.83	0.85	0.86	0.78	0.788	0.79
C6-C7	0.82	0.83	0.835	0.77	0.78	0.79
C7-T1	0.81	0.835	0.841	0.76	0.762	0.767

Table S3. Geometric changes of cervical discs under three postures.

Variable	Disc height	Disc height	Disc height	Cross-sectional area	Cross-sectional area	Cross-sectional area	Disc volume	Disc volume	Disc volume
Unit	mm	mm	mm	mm^2	mm^2	mm^2	mm^3	mm^3	mm^3
Posture	NBP1G	SM0G	NBP0G	NBP1G	SM0G	NBP0G	NBP1G	SM0G	NBP0G
C2-C3	3.1	3.13	3.15	380	390	392	1178	1220.7	1234.8
C3-C4	3.51	3.56	3.6	420	440	442	1474.2	1566.4	1591.2
C4-C5	4.12	4.2	4.21	490	512	515	2018.8	2150.4	2168.15
C5-C6	5.01	5.2	5.25	530	542	547	2655.3	2818.4	2871.75
C6-C7	4.2	4.27	4.28	540	570	577	2268	2433.9	2469.56
C7-T1	3.7	3.75	3.8	370	390	394	1369	1462.5	1497.2

Table S4. Ligament forces under three postures.

Variable	Ligament forces	Ligament forces	Ligament forces
Unit	N	N	N
Posture	NBP1G	SM0G	NBP0G
ALL	38.46	95.02	92.76
PLL	59.95	174.21	183.26
LF	41.86	89.367	85.97
